# Glomus jugulare tumors treatment by gamma knife radiosurgery: A single center study

**DOI:** 10.12669/pjms.39.1.6590

**Published:** 2023

**Authors:** Aurangzeb Kalhoro, Abdul Sattar M. Hashim

**Affiliations:** 1 Dr. Aurangzeb Kalhoro, FCPS, FACS Neuro Surgery. Consultant Neurosurgeon, Neuro Spinal & Cancer Care Institute, Karachi, Pakistan, Assistant Professor of Neurosurgery, Liaquat University of Medical & Health Sciences, Jamshoro, Sindh, Pakistan; 2 Prof. Dr. Abdul Sattar M. Hashim, PhD., M.D Neuro Surgery. Ex Prof JPMC, Medical Director, Neuro Spinal & Cancer Care Institute, Karachi, Pakistan

**Keywords:** Glomus Tumor, Gamma Knife, Stereotactic Radiosurgery

## Abstract

**Objective:**

Glomus jugulare tumor are benign vascular tumors and surgical resection is almost impossible. We have treated these tumors by Gamma knife radiosurgery and share our experience.

**Methods:**

This study was conducted at the Neurospinal and Cancer Care Institute, Karachi from January 2010 to May 2020. Thirty-four patients with glomus jugulare tumors treated with gamma knife radiosurgery were included in the study. The comprehensive clinical and demographic characteristics of all patients were collected through a manually designed questionnaire. Computed tomography, digital subtraction angiography and magnetic resonance imaging were used to make the diagnosis. Data was incorporated and analyzed by SPSS version 26.

**Results:**

A total of 34 patients were included in the study of which 16(47%) were males and 18(53%) were females with first follow up after 6-month up to two year clinical and radiological follow-up. The mean age of the patients was 42.5±13.5 with a minimum age of 20 years and maximum age of 65 years. The KPS scale was 2.09±0.45 and the volume of the tumor was 33.8±22.5 cm^3^. The improvement was shown in 27 patients of which 14 were males and 13 were females showing insignificance post radiation change. Of all 34 patients, the outcome was recorded as 3(9%) for excellent, 22(64%) for good, 6(17%) for fair and 3(9%) were poor results.

**Conclusions:**

Gamma Knife radiosurgery is a safe and effective primary therapy and salvage therapy for residual and recurrent cases of glomus jugulare and tympanicum tumors.

## INTRODUCTION

Glomus jugulare is a head and neck neuroendocrine benign lesion in nature and is a neural crest variant that occurs in the jugulare foramen. The location of glomus jugulare lies in the temporal bone of the skull base in the jugulare fossa.^1^,^2^ Occurrence of Glomus jugulare has been estimated to be between one and three per 100,000 people^1^ and are divided into two types: sympathetic and parasympathetic,^3^ they arise from the paraganglia cells located in the adventitia wall of the jugular bulb, within the jugular foramen. They grow slowly, however, as they grow in size, they can cause a mass effect by invading and eroding the temporal bone due to their position. Just 1% to 5% of all tumors are malignant.^2^ Adults between the ages of 40 and 60 are more likely to develop these tumors, with a slight female tendency.^4^

The most common symptoms are pulsatile tinnitus and conductive hearing loss. Ear fullness, otorrhea, hemorrhage, bruit, and the formation of a middle ear mass are other aural signs and symptoms. Vertigo and sensorineural hearing loss are caused by inner ear involvement.^5^ Glomus jugulare tumors have a difficult treatment problem because they penetrate major vessels and involve cranial nerves, resulting in severe morbidity from tumor resection.^6^ Because of age or physical condition, surgery might be associated with increased risk and large tumors affecting the lower cranial nerves and spreading past the petrous apex particularly in older patients, causing of postoperative complications related to surgery. Embolization, radiation, Gamma Knife stereotactic radiosurgery, or intratumoral cyanoacrylate glue injection can be considerable in such situations.^7^-^9^

The best treatment plan for Glomus Jugulare Tumor is still being discussed widely. It is very difficult to obtain the comparison of different treatment modalities because of the complexity and rarity of these kinds of tumors and other factors such as deficiency of prospective randomized trials as well as various selection conditions in retrospective studies for the specific treatment.^10^ The safe treatment option for glomus jugulare tumor is radiosurgery without any serious morbidity and mortality. The growth rate of these tumors is naturally slow. To determine a cure rate of these tumors after radiosurgery, a follow up of approximately ten years will be required.^11^

The abnormalities in the brain such as blood vascular malformations, well-delineated benign and malignant tumors and other psychiatric disorders can be treated by radiation such as Gamma Knife radiosurgery. This procedure is very different and less risky from other traditional surgeries.^6^ The successful rate of Gamma Knife radiosurgery is more than 90% in shrinking and stopping the growth of brain tumors. Gamma knife radiosurgery may take one to five sessions. Gamma knife radiosurgery is noninvasive method and safe method to treat highly vascular lesions of glomus jugular.^12^,^13^ For tumor regulation, the doses of marginal radiation might be ideal for greater than 13 Gy. The extensive follow-up will help researchers to understand the advantages and drawbacks of stereotactic radiosurgery better in the treatment of Glomus Jugulare Tumor patients.^14^ The rationale of the study is to observe the effect gammaknife radiosurgery in jugulare tumor which are highly vascular, surgical result may vary from patient to patient, so options like Gammaknife that are noninvasive method should be opted in patients and specifically such study on Gammaknife is not available is South Asia.

## METHODS

This was a retrospective descriptive study and was conducted from January 2010 to May 2020 at Neurospinal and Cancer Care Institute, Karachi. Total 34 patients with glomus jugulare tumors were treated with gamma knife radiosurgery. The patients gave written and verbal consent. Study was conducted after getting approval from the Institutional Research and Ethical Committee of Neuro Spinal Cancer Care Institute, Karachi (IRB: 9721/18, Dated: August 15, 2018). The comprehensive clinical and demographic characteristics of all patients such as age, gender, symptoms were collected through a manually designed questionnaire. Histological findings led to the diagnosis of GJT. CT (computed tomography), followed by MRI (magnetic resonance imaging) were done in all patients to exclude any other pathology and making diagnosis more accurate DSA (digital subtraction angiography), was done that also helped in planning.

### Radio Surgical Procedures:

Gamma knife 4C Model and Leksell Gamma Knife icon model was used for the treatment over the specific area within specified time. Local anesthesia was applied for the Leksell frame which was fixed and was followed by the 1-mm- thin MRI sequences. The dose was planned by a team having a neurosurgeon, medical physicist and neuroradiologist. The mean volume of the tumor was 33.8±22.5cm^3^. The median tumor dose was 17.4 Gy (range 13–25 Gy) and it was the 50% isodose line (range 40%–56%). In our setup all patients were done in single session, while based on Glasscock and Jackson classification, the Glasscock– Jackson classification, in which the Grade I is a small tumor that involves the jugular bulb, middle ear, and mastoid, the Grade II can be the tumor that extends under the internal auditory canal while Grade III is the one lesion extends in the petrous apex; while the Grade-IV is said to be the one which extends further than petrous apex and involving the clivus or infratemporal fossa^13^ while in our study, Grade-I we had 6 (17.6%) tumors, Grade-II we had 9 (26.4%), in Grade-III we had 10 (29.4%) and Grade-IV we had 9 (26.4%) tumors.

### Statistical Analysis:

Data was incorporated and analyzed by SPSS version 26. Demographic data such as age, KPS scale, volume and prescription details were presented as mean and standard deviation and frequency along with their percentages were employed for categorical variables such as gender, histopathological details, history of radiotherapy and others. Paired sample t-test was applied for the volume of tumor before and after surgery. Continuous variables were analyzed by t-test. Chi-square was applied to determine the association of outcome variables of gamma knife surgery with baseline characteristics. The p-value less than 0.05 was considered a significant level.

## RESULTS

A total of 34 patients were included in the study in which 16(47%) were males and 18(53%) were females. The mean age of the patients was 42.5±13.5 with minimum age of 20 years and maximum age of 65 years, as shown in Table-I. The patients were followed up after every six months up to two years of clinical and radiological assessment. The KPS scale was 2.09±0.45 and the volume of the tumor was 33.8±22.5cm^3^. Out of 34 patients, 6(18%) patients had open prior biopsy and stereotactic and only one patient had the history of radiotherapy. The demographic characteristics of all the patients were shown in Table-II. Grade-I we had 6 (17.6%) tumors, Grade-II we had 9 (26.4%), in Grade-III we had 10 (29.4%) and Grade-IV we had 9 (26.4%) tumors

**Table-I:** Association of outcome with baseline characteristics.

	Values Improvement		p-value

	Yes	No	
Gender*			0.271
Male, n (%)	14 (52)	2 (28)	
Female, n (%)	13 (48)	5 (71)	
Age Group*			0.071
≤50 years, n (%)	21 (78)	3 (43)	
>51 years, n (%)	6 (22)	4 (57)	

* Fisher exact test was applied.

**Table-II:** Demographic Characteristics (n=34).

Parameter	Values
Age(years), mean±SD	42.59±13.5
***Gender***	
Male, n (%)	16 (47%)
Female, n (%)	18 (53%)
KPS Scale, mean±SD	2.09±0.4
Overall Volume, mean±SD	33.8±22.5
Volume after treatment, mean±SD	22.7±19.8
***Improvement***	
Yes, n (%)	27 (79%)
No, n (%)	7 (21%)

SD: Standard Deviation.

Primary cases were 28 patients (82.3%) and secondary cases were 6(17.6%) patients. The skull base tumor, glomus jugulare tumors, usually occurs between the lower part of the brain that is cerebelleum and the lower cranial nerves. All glomus jugulare tumors were benign tumors in the study, 23(67.6%) were located on the right and 11(32.3%) were on right. The mean volume of the tumor was significantly low after the treatment (p-value <0.001).

The overall improvement was seen in 27 patients in which 14 were males and 13 were females showing insignificance (p-value=0.271). Of all 34 patients, the outcome was recorded as 3(8.4%) for significant regression in lesion, 22(64.7%) for improved with moderate regression, 6(17.6%) for mild improvement and 3(8.8%) did not respond well to treatment, based on clinical improvement of the patient as mentioned in Table-III.

**Table-III:** Improvement status of the prior symptoms

Symptoms	Prior Symptoms	Improvement (n)	

		Yes	No
Headache, n (%)	9 (27%)	8	1
Earache, n (%)	4 (12%)	3	1
Vertigo, n (%)	11 (32%)	9	2
Dysphagia, n (%)	3 (9%)	3	0
Facial Numbness, n (%)	8 (24%)	7	1
Tinnitus, n (%)	15 (44%)	12	3
Difficulty in chewing/swallowing, n (%)	9 (27%)	8	1
Blur vision, n (%)	2 (6%)	2	0
Hearing of voice, n (%)	6 (17%)	3	3
Loss of hearing, n (%)	13 (38%)	12	1

## DISCUSSION

Until the end of the past decade, the only therapeutic options for glomus jugulare tumors were microsurgical excision, conventional radiation therapy, endovascular embolization and a blend of these. However, when efficacy of GK therapy was established in the treatment of highly vascular brain tumors and cerebral arteriovenous malformations, the nature of paragangliomas made them suitable marks for gamma knife radiosurgery. Gamma Knife surgery has fewer problems with a petite duration of intervention and hospital stay than traditional radiation. GKS has a low risk of complications due to the ionization of a small quantity of tissue.^15^-^17^

In this study the proportion of improvement in the patients treated by gamma knife radiosurgery was high up to 79%. Eustacchio S et al published after neurological follow up that the clinical status was improved among the patients and no complication occurred. They stated that the gamma knife radiosurgery is a viable therapeutic option for Glomus jugulare tumors.^18^

Genc A et al showed in their study that the radiosurgery is a safe and effective therapeutic method for the therapy of glomus jugulare tumors.^6^ It satisfies the needs of both the surgeon as well as the patients, providing more relief with less complication after the procedure. They performed the analysis on the series of patients with glomus jugulare tumors treated with gamma knife radiosurgery, and they followed the longest mean follow-up during their research period. They also suggested that the GKRs is a viable and safe therapeutic option for not surgically treated glomus jugulare tumors.^6^

Our study showed significant decreased of tumor size from 33±3 to 22±3 on average in all patients. Gottfried et.al in an investigation of eight series of stereotactic radiosurgery observed the decreased tumor size in 36.5 percent of patients. Sixty-one percent of the individuals studied showed no change in tumor size, whereas 2.2 percent had increased tumor development.^13^ Treatment at an early stage, before tumors get large size, may prevent post-treatment problems.

Since 1999, the radiosurgery has been promoted as an alternate therapy for glomus jugulare tumors. Lee CC et al showed that the tumor regression rate was the highest among others when compared to other series. Glomus tumors are known as the particularly radiosensitive, with control rates of tumor ranging from 86-97 percent ensuing standard radiation, according to many studies. However, there were indications of the live tumor cells after radiation, suggesting that radiation’s long-term response is uncertain. The results of their research showed that the Gamma Knife radiosurgery of fourteen patients with tympanicum and glomus jugulare had a strong tumor with a control rate of 100% and a high cranial nerve preservation rate of 92.7% after the follow-up with a median of 40.3 months. For patients with glomus jugulare tumor and tympanicum, the substantial protection of lower cranial nerve function, in particular, enhanced their life quality. They suggested the gamma knife surgeries may be a worthwhile option to surgery, either as adjuvant therapy or as a main treatment following inadequate surgery.^19^

Ibrahim R et al contributed to the research by establishing steady outcomes of long-term following gamma knife radiosurgery. For remaining and recurring cases of tympanicum and the glomus jugulare tumors, the most effective, standard and survival therapy is the Gamma Knife radiosurgery. It provides low risk-versus-benefit therapy along with long-term controlling rates of the tumor that are steady with the few adverse effects.^9^ With no mortality and no severe morbidity, the gamma knife radiosurgery has proven to be a safe therapy for glomus jugulare tumors. Because of the sluggish development rate of these lesions, up to ten years of follow-up will be required to determine a cure rate after radiosurgery.^11^

### Limitations:

Number of patient will increase with time, long term follow up, our study had patient with effect of purely treatment based on gammaknife while those with pre or post radiation surgery can be added in future study to see its effect also.

## CONCLUSION

Gamma Knife radiosurgery is a safe and effective primary therapy and salvage therapy for newly diagnosed and recurrent cases of glomus jugulare and tympanicum tumors. It offers a low risk-benefit treatment option with stable long-term tumor control rates and minimal side effects. Treatment at an early stage before tumors reach large sizes potentially reduces post-treatment complications.

### Authors’ Contribution:

**AK:** Conception, design of study, acquisition of data, Statistical analysis, interpretation of data..

**ASMH:** Prepared the first draft, critical analysis and intellectual input besides Final approval of manuscript.
